# OntoPESScan: An Ontology for Potential Energy Surface Scans

**DOI:** 10.1021/acsomega.2c06948

**Published:** 2023-01-03

**Authors:** Angiras Menon, Laura Pascazio, Daniel Nurkowski, Feroz Farazi, Sebastian Mosbach, Jethro Akroyd, Markus Kraft

**Affiliations:** † Department of Chemical Engineering and Biotechnology, University of Cambridge, Philippa Fawcett Drive, Cambridge CB3 0AS, U.K.; ‡ CARES, Cambridge Centre for Advanced Research and Education in Singapore, 1 Create Way, CREATE Tower, #05-05, Singapore 138602; ¶ CMCL Innovations, Sheraton House, Castle Park, Cambridge CB3 0AX, U.K.; § School of Chemical and Biomedical Engineering, Nanyang Technological University, 62 Nanyang Drive, Singapore 637459; ∥ The Alan Turing Institute, London NW1 2BD, United Kingdom

## Abstract

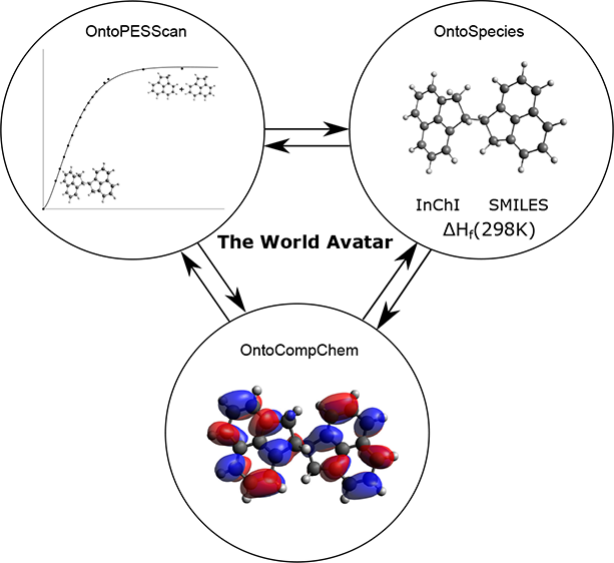

In this work, a new OntoPESScan ontology is developed for the semantic representation of one-dimensional potential energy surface (PES) scans, a central concept in computational chemistry. This ontology is developed in line with knowledge graph principles and The World Avatar (TWA) project. OntoPESScan is linked to other ontologies for chemistry in TWA, including OntoSpecies, which helps uniquely identify species along the PES and access their properties, and OntoCompChem, which allows the association of potential energy surfaces with quantum chemical calculations and the concepts used to derive them. A force-field fitting agent is also developed that makes use of the information in the OntoPESScan ontology to fit force fields to reactive surfaces of interest on the fly by making use of the empirical valence bond methodology. This agent is demonstrated to successfully parametrize two cases, namely, a PES scan on ethanol and a PES scan on a localized π-radical PAH hypothesized to play a role in soot formation during combustion. OntoPESScan is an extension to the capabilities of TWA and, in conjunction with potential further ontological support for molecular dynamics and reactions, will further progress toward an open, continuous, and self-growing knowledge graph for chemistry.

## Introduction

The potential energy surface (PES) is one of the key concepts of computational chemistry, representing the relationship between the energy of a molecule or system of molecules and the geometry or coordinates of said system.^1^ The potential energy surface thus allows representations and changes in the shape and geometry of a molecule or system of molecules to be related to the system electronic energy, enabling the application of Schrödinger’s equation to molecules. As a consequence, the determination and descriptions of potential energy surfaces find a wide variety of use in computational chemistry. This includes the accurate computation of rate coefficients of chemical reactions, as chemical reactions are naturally represented by the potential energy surface and a good description of the PES is necessary to compute the rate of the reaction described by the surface.^2^ Potential energy surfaces are also central to the development of force fields for molecular dynamics simulations, which must capture how interactions between different chemical species result in changes in system energies and thus require a representation of the underlying PES.^3^ Achieving this all requires chemical data on potential energy surfaces, reactions, and the chemical species they describe.

An increasing amount of computational chemical information is stored in online databases for the purpose of sharing information.^4^ Examples include the Computational Chemistry Comparison and Benchmark DataBase (CCCBDB) from the National Institute of Standards and Technology (NIST), which houses energetic, vibrational, and thermochemical information for a variety of species determined by various initio quantum chemistry methods,^5^ and the Alexandria library of calculations for force field development.^6^ Well-known general chemistry databases such as Pubchem^7,8^ also provide information on cheminformatics identifiers to uniquely define and link different data on chemical species as well as geometry and crystal structure information relevant to computational chemistry efforts. Information science and mathematical methods such as graph theory and machine learning are increasingly used with such chemical data to further progress, with examples seen in organic reaction network analysis,^9,10^ predictive combustion chemical kinetics,^11^ the use of machine learning to suggest retrosynthetic pathways in Reaxys,^12,13^ and the application of machine learning to fit potential energy surfaces.^14^

A key group of methods that facilitate the access and manipulation of such chemical data are Semantic Web technologies. Semantic Web approaches such as knowledge graphs (KGs) and ontologies provide frameworks for the storage, representation, and annotation of information in an consistent and well-defined manner. They also allow for clear logical approaches in the querying and manipulation of data and have been seeing increasing use in chemistry. Some key examples include the chemical information ontology,^15^ the chemical ontology built on methontology,^16^ the ChEBI ontology for information on chemical species of biological interest and applications,^17−19^ and PubChem’s RDF representation of its data and annotations.^8,20^ Additionally, there are several chemistry-related ontologies as part of the dynamic cross-domain knowledge graphs comprising the J-Park simulator (JPS) and The World Avatar (TWA) projects.^21,22^ The World Avatar is discussed in detail later, but briefly the chemistry ontologies include OntoCompChem for representing quantum chemistry calculations,^23^ OntoSpecies for representing chemical species,^24^ OntoKin for representing chemical reaction mechanisms,^25^ and OntoChemExp for representing chemical experiments.^26,27^

The purpose of this paper is to extend the chemistry section of The World Avatar knowledge graph by developing and introducing an ontology for the representation of potential energy surfaces, OntoPESScan. In its first implementation, OntoPESScan is developed to represent one-dimensional (1D) PES scans along a scan coordinate. This is a substantial approximation because it only represents a portion of the full multidimensional surface. However, 1D scans, also known as linear transits, are a widely used in many applications, from the use of 1D rotor scans to handle the treatment of conformers and low-magnitude vibrations in the partition function to approaches where a collection of linear transits are used to construct force fields to allow simulations of larger systems of interest. These 1D methods are often used as computationally tractable approaches where the calculation of full-dimensional potential energy surfaces (of dimension 3*N* – 6, where *N* is the number of atoms) is not feasible for high-throughput approaches.

The development of OntoPESScan is meant to maximize the reuse of computational chemical data generated by various researchers by providing a framework for the data to be stored in a consistent and semantic way. As an example of this, a force-field agent is also developed to fit a force field based on the chemical data in OntoPESScan. The construction of the force-field agent reported here demonstrates proof-of-concept and serves to show how the knowledge graph framework can further progress data automation in chemistry, as well as the representation and analysis of potential energy surfaces and force fields that find wide applications in chemistry. It is worth noting that OntoPESScan is the first ontology that has frameworks for describing potential energy surfaces and connects to semantic descriptions of chemical species and computational chemistry calculations. Additionally, although it is currently limited to the representation of 1D PES scan, there is scope for expansion. Nevertheless, OntoPESScan serves an example of how key chemical concepts can benefit from semantic approaches.

## The World Avatar

### Overview

The World Avatar is built using a dynamic knowledge graph (dKG) that makes use of linked data principles and the semantic web to represent concepts as nodes of a graph and relationships between said concepts as vertices of a graph. The linking of information is important because it enables concepts and information across different domains to be linked together. The World Avatar makes use of ontologies to define the relationships and key concepts for a given domain in what is known as the terminological component or TBox. Instances, data, and facts about the concepts and relations in the TBox form the assertion components or ABox. These are often instantiated in a triple store, which contains the formal subject–predicate–object relations for the data.

Agents are key aspects of the dynamic knowledge graph. Agents are autonomous bits of software that continuously and independently act on the knowledge graph, leveraging the data and structure of the KG to perform various tasks. Such tasks include performing calculations using data in the KG; passing information to other software or users outside of the KG and then taking these results to create new instances (ABoxes) in the KG; updating existing instances in the KG with improved information where appropriate; and updating the concepts, definitions of concepts, and relationships between concepts in the Ontological TBoxes. Agents perform these tasks with the aim of producing a self-growing, self-updating, and self-improving knowledge graph. Additionally, agents can also compose composite agents to answer more complex queries or perform more complex tasks. Agents themselves are integrated as part of The World Avatar knowledge graph by means of an ontology that semantically describes agents, namely, OntoAgent,^28^ and a market for using and identifying new agents.^29^

**Figure 1:**
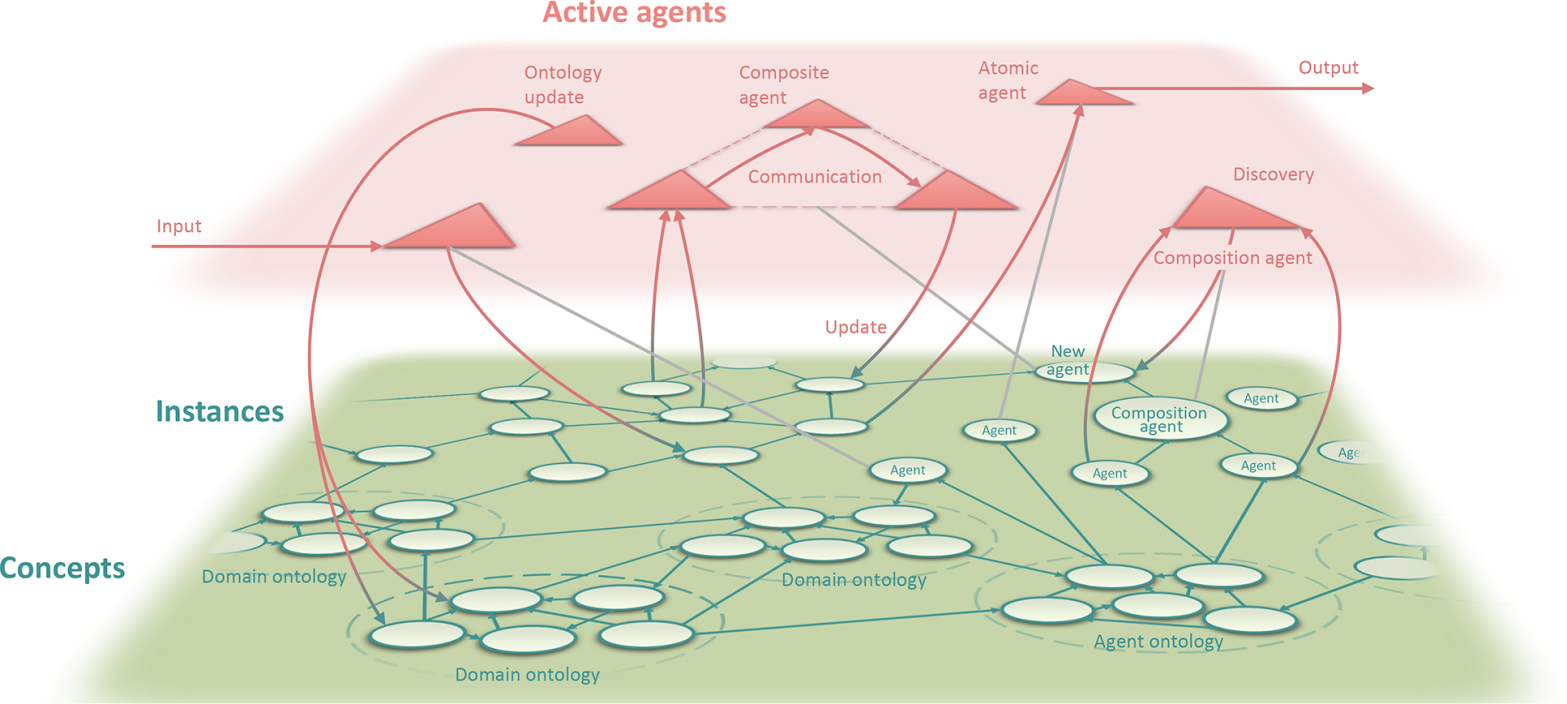
Structure of The World Avatar knowledge graph. Reprinted with permission from ref (30) under a CC BY 4.0 license. Copyright 2021 Cambridge University Press.

Ontologies, instances, and agents form the main components of the knowledge graph, as illustrated schematically in Figure 1.

The World Avatar KG currently includes several ontologies that span a variety of domains. The ontologies included in the chemistry domain (OntoKin,^25^ OntoSpecies,^24^ OntoCompChem,^23^ and OntoChemExp^26^) are discussed in detail in the next section.

Several examples of agents and cross-domain applications also exist in TWA. This includes the thermochemistry agent that determines thermochemical parameters for chemical species using information in OntoSpecies, which has been combined with agents for modeling engines using kinetics in OntoKin and an Atmospheric Dispersion Agent to demonstrate how underling chemical mechanisms in ship engines can impact predicted pollutant distributions in nearby cities.^31^ Additional agents include those that can predict the power conversion efficiency of organic solar cells based on SMILES strings and organic donor properties in OntoSpecies in conjunction with machine learning approaches.^32^ There are also several links between the chemistry domain ontologies, which are discussed further below.

### Chemistry in The World Avatar

This section summarizes the current main ontologies and semantic web technologies in TWA that support the chemistry domain.

#### OntoSpecies

The OntoSpecies ontology serves as a chemistry ontology, with entries in the ontology consisting of unique chemical species and chemical key properties. This is in line with other major chemistry resources such as Pubchem,^8^ for example. The full ontology is found at http://www.theworldavatar.com/ontology/ontospecies/OntoSpecies.owl. Each entry in OntoSpecies is assigned a unique Internationalized Resource Identifier (IRI). This allows the IRI to uniquely identify a chemical species and enables a chemical species and its associated information to be linked to instances and concepts in other related ontologies. Basic properties represented in OntoSpecies include semantic descriptions of molecular formula, charge, molecular weight, and spin multiplicity. Isotopes, different charges, and different spin states are treated as different chemical species to the most common standard state. The standard enthalpy of formation is also represented in OntoSpecies, as this has use in several reactor simulations. The reference temperature, reference state, and provenance for the standard enthalpy of formation are also included in the ontology to add the necessary contextual information.

OntoSpecies also includes concepts for key cheminformatics identifiers widely used in the field, namely, InChI and its associated hash representation (InChIKey) from IUPAC^33^ and SMILES.^34,35^ This allows users to search for entries in OntoSpecies by their InChI or SMILES codes. Other identifiers supported in OntoSpecies include those from major chemistry databases in PubChemCID for PubChem and CASID for the Chemical Abstracts Service where available. This facilitates searching for additional information on a chemical species in these external resources if it is not found in OntoSpecies. The final concept category in OntoSpecies consists of geometric properties of a species. This includes a list of bonds in the species as well as a semantic representation for the full three-dimensional geometry of a species, which serves as a curated reference geometry. This reference geometry serves two purposes. First, it provides unique IRIs for each atom in a chemical species, which means these atoms can also be uniquely identified in addition to just the overall chemical species. This can be important when comparing different quantum chemical calculations on the species, where the order of atoms is often flexible. Additionally, this geometry can also be used as a starting point for the aforementioned quantum chemical calculations for an agent. While it is possible for geometries to be derived from InChI and SMILES strings through format translators and molecular force fields, as implemented in OpenBabel,^36^ this approach can encounter issues when dealing with metals for example, so having a ready-out-of-the-box geometry can prove advantageous in such situations.

#### OntoKin

The OntoKin ontology is used to semantically represent chemical reaction mechanisms. The full ontology is published at http://www.theworldavatar.com/ontology/ontokin/OntoKin.owl and covered in detail in Farazi et al.^25^ The key concepts in OntoKin essentially cover those that are required to describe the most common chemical mechanism format, with CHEMKIN^37^ in particular being a main reference. Concepts in OntoKin include definitions of a chemical mechanism, which consists of a set of chemical reactions. Chemical reactions occur among different chemical species that consist of chemical elements, react in ratios defined by stoichiometric coefficients, can be reversible and irreversible. Additionally, the phase concept is also defined in OntoKin enable the representation of both gas phase and surface (solid) phase reactions. The rates of these reactions are represented using rate models (i.e., Arrhenius-type), which are used to compute rate coefficients. Thermodynamic and transport model concepts are also associated with species. Of note, multiple rate models can be associated with the same reaction, as can multiple thermodynamic or transport models with a given species. This allows OntoKin to support the facile comparison of different kinetic, thermodynamic, or transport models in the literature for the user.^24^ Additionally, species in OntoKin can be linked to a species instance in OntoSpecies, enabling a unique identification. This helps resolve naming inconsistencies of species across different chemical mechanisms, for example, where benzene could be called “A1” in one mechanism but “C_6_H_6_” in another.^24^

#### OntoCompChem

The OntoCompChem ontology is used to semantically represent the results and details of computational chemical calculations. The full ontology is published at http://www.theworldavatar.com/ontology/ontocompchem/ontocompchem.owl and covered in detail in Krdzavac et al.^23^ OntoCompChem includes concepts that cover the inputs and outputs of the most common computational chemistry calculations and builds on concepts defined in the Gainesville Core (GNVC) ontology.^38^ On the input side, this includes the level of theory used to perform the calculation in terms of the functional (i.e., B3LYP) and the basis set (i.e., 6-31G(d)), as well as the charge and multiplicity. These are inputs that virtually all computational chemistry packages require. OntoCompChem also includes classes that help define what program is used to perform the quantum chemical calculation. Currently, such classes are available for the widely used Gaussian programs Gaussian 09 and Gaussian 16^39−41^ but can easily expand to include other programs.

On the output side, the results of single-point energy, geometry optimization, and frequency calculations are represented. For energies, this includes the final converged self-consistent field (SCF) energy and, if an accompanying frequency calculation is present, the zero-point energy correction. Frontier orbital energies and near orbital energies are also represented, namely, the HOMO – 2, HOMO – 1, HOMO, LUMO, LUMO + 1, and LUMO + 2 energies. For geometry optimization calculations, a full 3D representation of the optimized geometry is present in OntoCompChem, with full coordinate values, atom types, and rotational constants all included. For frequency calculations, a full list of the computed vibrational frequencies are represented, enabling easy confirmation of the type of stationary point a geometry or calculation corresponds to. Finally, much like OntoKin, OntoCompChem calculations also contain a link to OntoSpecies in terms of the “hasUniqueSpecies” concept, which points to an IRI in OntoSpecies and defines what species the calculation was run on. This supports a clear way to group together different quantum chemistry calculations performed on the same species, making it straightforward for a user to compare how different methodologies impact the results.

#### OntoChemExp

The OntoChemExp ontology is developed to enable semantic representation of chemical experiments. The full ontology is published at http://theworldavatar.com/ontology/ontochemexp/OntoChemExp.owl and is covered in detail in Bai et al.^26^ Concepts were initially developed with the representation of combustion experiments in mind, but the four-modular structure is broadly applicable. This structure includes the experiment module, which includes the experiment instance and its associated metadata in terms of the source from which the experiment and its data were taken from. OntoChemExp also includes setup modules where details on the apparatus and key experimental conditions are represented, a results module where the data collected from the experiment is abstracted in terms of a data group for each independent variable and data points measured in each data group, and finally a specification module where concepts such as values and uncertainties are defined that can then be assigned to conditions or data instances in the setup or results section. As with OntoKin and OntoCompChem, the “hasUniqueSpecies” concept is present to enable the unambiguous identification of chemical species involved in experiments through the connection to a unique OntoSpecies IRI.

#### Marie

The World Avatar also includes the Marie Web site, which serves as a question-and-answer system for the chemical information in the KG.^29^ Marie was developed with the intention of lowering the barrier to interacting with the knowledge graph and various ontologies. One typically accesses information in the KG through query construction languages such as SPARQL.^42^ While SPARQL allows for logical and complex queries to be carried out, it can be a barrier for unfamiliar users, so Marie provides a natural question-and-answer approach to access through which users can request or search for information much like they would in a typical search engine such as Google or Wolfram Alpha. Marie makes use of natural language processing (NLP) techniques to map the question asked by the user to SPARQL queries that traverse the knowledge graph to find the information that answers the question. Marie can currently answer several chemistry-related questions, including finding kinetic and thermodynamic properties for species or finding out what reactions a species is involved in.

A recent extension also leverages the semantic framework for agents, where if a user’s question is not answerable purely based on the static data in the KG Marie will automatically identify and invoke an appropriate agent to try and derive an answer based on the information in the KG. The NLP framework also has an automated approach to help identify new question types that can be answered when a new agent is added in the KG. An example of this is calling the thermochemistry agent to calculate the thermodynamic properties of a chemical species requested by a user. Even if the thermodynamic properties are not explicitly stored in the KG, if the thermochemistry agent can identify an appropriate quantum chemical calculation for the requested species, it can calculate the required thermodynamic properties and return them to the user. As more instances, ontologies, and agents are added to TWA, Marie will automatically improve to answer a wider variety of questions from the user.

The World Avatar currently includes ontologies that can represent a variety of chemical concepts for species and their properties, reactions and kinetics, quantum chemistry calculations, and chemical experiments. However, the representation of potential energy surfaces is not included in the above ontologies and is a key concept in computational chemistry, the derivation of reaction rates, and the large field of molecular dynamics. Thus, OntoPESScan was developed to fill this gap while also naturally linking to existing ontologies.

## The OntoPESScan ontology

### Main Structure

**Figure 2:**
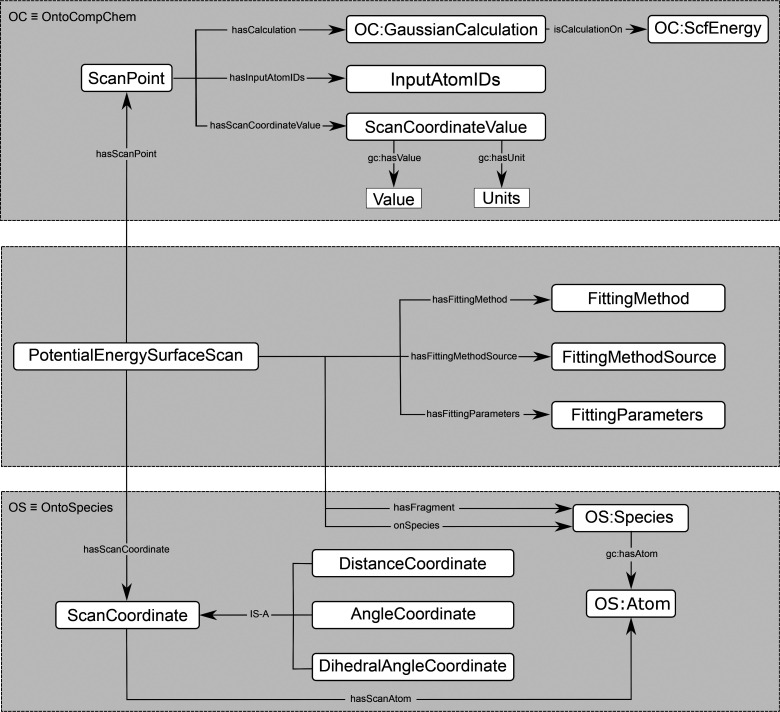
Structure of the OntoPESScan ontology developed in this work. The main classes and concepts are illustrated by boxes, with the relations between these classes represented by the arrows. Links to OntoSpecies (OS) and OntoCompchem (OC) are also shown.

The OntoPESScan ontology was developed to be a compact representation of the key concepts necessary to semantically represent scans and explorations of potential energy surfaces. Currently, OntoPESScan describes one-dimensional potential energy surfaces arising from scans along a bond, angle, or dihedral coordinate. This is a substantial dimension reduction, so the current framework is most suited for representing small molecular systems. The linked data principles of the knowledge graph are used as guidelines so that information is not unnecessarily duplicated in this new ontology. This is achieved by linking the OntoPESScan ontology to existing chemistry ontologies in The World Avatar, namely, OntoSpecies and OntoCompChem. The structure of the OntoPESScan ontology is shown in Figure 2. The full terminological component (TBox) of the ontology containing the full class and relational definitions is available at http://theworldavatar.com/ontology/ontopesscan/OntoPESScan.owl.

Figure 2 shows that the OntoPESScan TBox broadly contains three main conceptual sections. The first in the center contains the main class of the ontology namely “PotentialEnergySurfaceScan”. This class is used to define instances of scans on potential energy surfaces in the ontology. Connected to this class are three data properties, namely, “hasFittingMethod”, “hasFittingMethodSource”, and “hasFittingParameters”, which each connect the “PotentialEnergySurfaceScan” instance to string instances that provide contextual descriptions of the method or methods that are used to fit the potential energy surface scan instance. An example of this would be fitting a Morse potential to an intermolecular interaction, as is often done when using potential energy surface scans to compute the rate of reaction between two radicals in models such as the Gorin Model.^43^ In this case, the “hasFittingMethod” data property would link the “PotentialEnergySurfaceScan” instance for the reaction of interest to “MorsePotential”, with the “hasFittingMethodSource” being assigned to a specific publication or reference that describes the Morse Potential method in detail. The “hasFittingParameters” would then be the Morse Potential parameters, typically *D*_e_ for the well depth, *r*_e_ for the equilibrium bond distance, and *a* for the width of the potential well, and their values.

The next key relations are object properties that connect instances of the “PotentialEnergySurfaceScan” to instances of Species in the OntoSpecies ontology. These are the “onSpecies” and “hasFragment” object properties. The “onSpecies” object property enables a user to query the scans in OntoPESscan using what species the scan was performed on and makes use of the fact that OntoSpecies species IRIs are unique to resolve any ambiguities. This is an identical approach to what is adopted in OntoCompChem, OntoKin, and OntoChemExp, as discussed previously. The “hasFragment” property is similar to “onSpecies” but accounts for the fact that a potential energy surface can involve multiple species. Common examples of this would include bond-forming processes between two radicals or the reverse process of bond breaking in a chemical species that results in two species as products. As a result, the “PotentialEnergySurfaceScan” class needs to be able to be semantically linked to multiple OntoSpecies Species, and the “hasFragment” object property enables this. Although only 1D potential energy surface scans can be currently stored in OntoPESScan, multiple scans on different scan coordinates or performed using different methods can be linked to the same species. This allows easy storage and access to all the information on a specific species if needed. The main goal of OntoPESScan is indeed to facilitate the storage and retrieval of data for these type of calculations that find a wide range of applications.

### Scan Coordinates

The second main conceptual section in Figure 2 concerns the definition of the scan coordinate, which is essentially the geometric variable with which the energy of the chemical system varies along the potential energy surface. The central class is thus “ScanCoordinate”, and instances of this class are linked to instances of “PotentialEnergySurfaceScan” through the object property “hasScanCoordinate”. Three subclasses of scan coordinate are defined in the OntoPESScan TBox, inheriting the properties of “ScanCoordinate” through the IS–A relation. These are “DistanceCoordinate”, “AngleCoordinate”, and “DihedralAngleCoordinate”, where each of these coordinates defines a different geometric type of scan. Currently, the “PotentialEnergySurfaceScan” instance is linked to only one “ScanCoordinate” instance, allowing the representation of one-dimensional potential energy surfaces. Such scans are used throughout the computational chemistry literature, for example, in hindered rotors.^44^ Nevertheless, it is important to note that the full dimensionality of the PES is 3*N* – 6, where *N* is the number of atoms, and 1D representations are substantial reductions from the full potential energy surface. However, the ontology can support simple representation of multidimensional surfaces by linking the “PotentialEnergySurfaceScan” instance to multiple “ScanCoordinate” instances. Further extensions to include more complex multidimensional coordinates would also assist with multidimensional surface representation but are left to further work.

**Figure 3:**
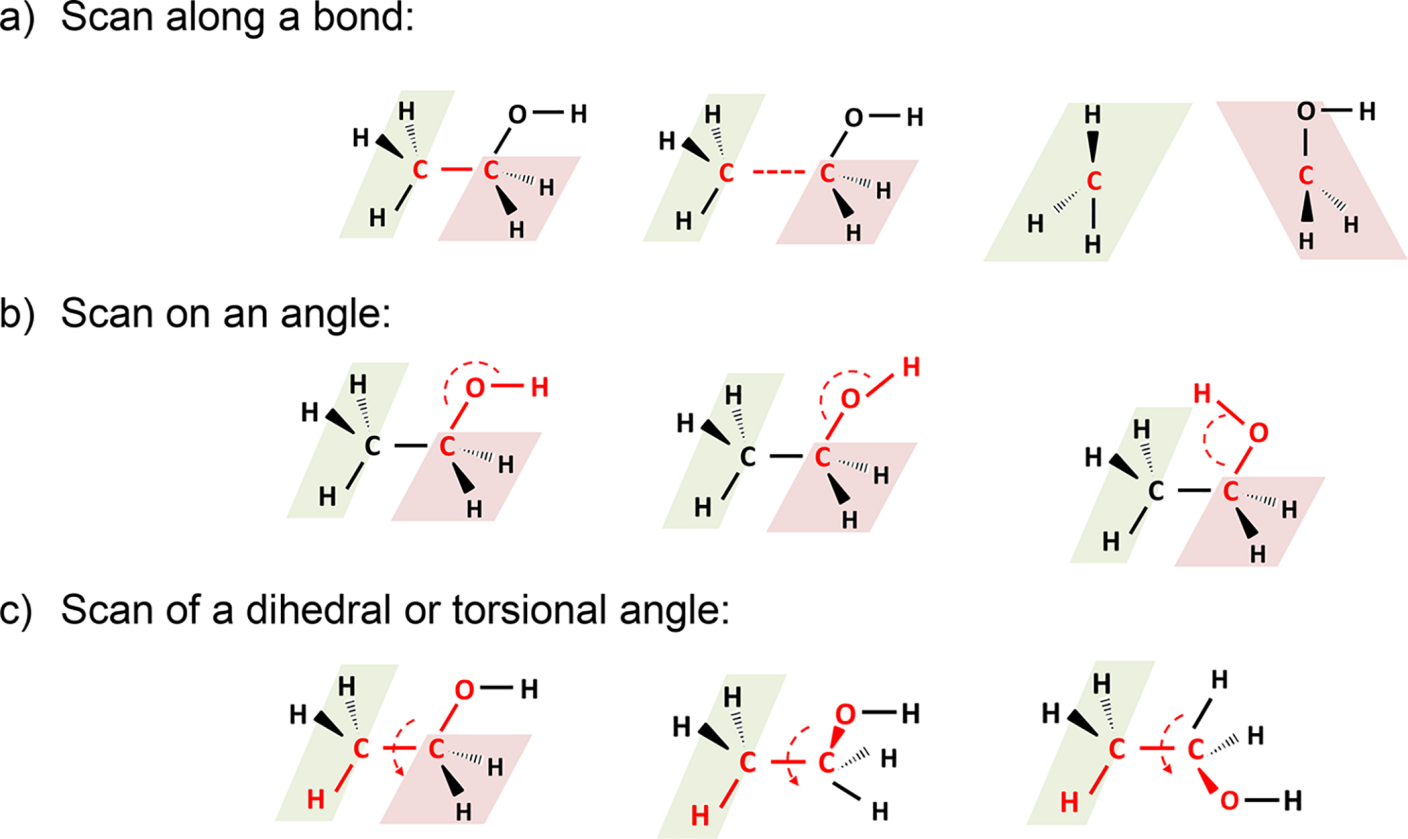
Examples of the three main different types of scans represented semantically in OntoPEScan, as shown for ethanol. This includes (a) bond scans, (b) plane angle scans, and (c) dihedral angle scans. The atoms that define the scan coordinate are displayed in red.

Figure 3 illustrates three different scans on an ethanol molecule that correspond to the three scan coordinate subclasses defined in OntoPESScan. The first is a potential energy surface derived from a scan along a bond, with the scan coordinate in this case being the carbon–carbon bond length in an ethanol molecule. This would be defined by a “DistanceCoordinate” instance. The “DistanceCoordinate” instance would then need to be defined in terms of the atoms that comprise this coordinate, namely, the two carbon atoms in ethanol. To uniquely define the scan coordinate, OntoSpecies is again used, with instances of the “ScanCoordinate: class being linked to atom IRIs in OntoSpecies through the “hasScanAtom” object property. Using IRIs, the problem of different users uploading variations of the same scan on ethanol but with different orderings of atoms in their computational calculations is circumvented. This is because there is a unique instance for ethanol in OntoSpecies, with each atom having its own unique IRI and fully defined coordinates, enabling the unique identification of the atoms. Such bond scans have a variety of applications, being necessary to compute rate constants for bond-forming or bond-breaking reactions for example, as shown in Smith and Golden.^43^ They are also crucial when developing reactive force fields such as ReaxFF,^45^ where they are used as references for fitting the necessary force-field parameters that can then be applied to larger systems of molecules.

The second type of scan is a potential energy surface derived from a scan along a plane angle, in this case the angle formed by the carbon–oxygen bond and the oxygen–hydrogen bond in ethanol, as illustrated in in Figure 3b. This would be represented by an “AngleCoordinate” instance, which would be connected to the appropriate carbon, oxygen, and hydrogen atom in OntoSpecies through the hasScanAtom object property as with a bond scan. This is a less commonly used type of scan but is necessary for parametrizing bond-bending and flexural interactions in reactive force fields,^45^ and angle changes do occur in organic dehydration and dehydrogenation mechanisms. The third type of scan corresponds to a potential energy surface derived from a scan along a dihedral or torsional angle, in this case illustrated for the hydrogen–carbon–carbon–oxygen dihedral in ethanol. This would be represented by a “DihedralAngleCoordinate” instance, with four atoms from the OntoSpecies entry for ethanol being connected to the instance in OntoPESScan in this case, akin to the previous bond and plane angle cases. Dihedral and torsional angles are also widely used, again being crucial for parametrizing reactive force fields but also for deriving potential energy surfaces and rate constants for cis–trans isomerization reactions, as shown in Figure 3c for ethanol. Fittings of dihedral angle scans are also used to apply hindered rotor corrections to partition functions when computing rate constants, which is seen in several rate coefficient determining computer programs such as MultiWell,^46^ Reaction Mechanism Generator,^47^ and VaReCoF.^48^

### Scan Points along the PES

The third main conceptual section in Figure 2 concerns the definition of the scan point. Here, an instance of the “ScanPoint” class is connected to an instance of “PotentialEnergySurfaceScan” through the “hasScanPoint” object property, allowing for each point on a potential energy surface to be its own instance while having a common association with a single scan. Each scan point is connected to an instance of the “ScanCoordinateValue” class through the “hasScanCoordinateValue” object property. The “ScanCoordinateValue” instance is then linked to associated value and unit through data and object properties borrowed from the GainesVilleCore ontology^38^ and unit definitions from NASA’s QUDT ontology.^49^ For example, the first scan point along the carbon–carbon bond scan in ethanol would have its own instance linked to a “ScanCoordinateValue” instance, which would then have a value of 1.51 and a unit of the Ångström class from QUDT.

**Figure 4:**
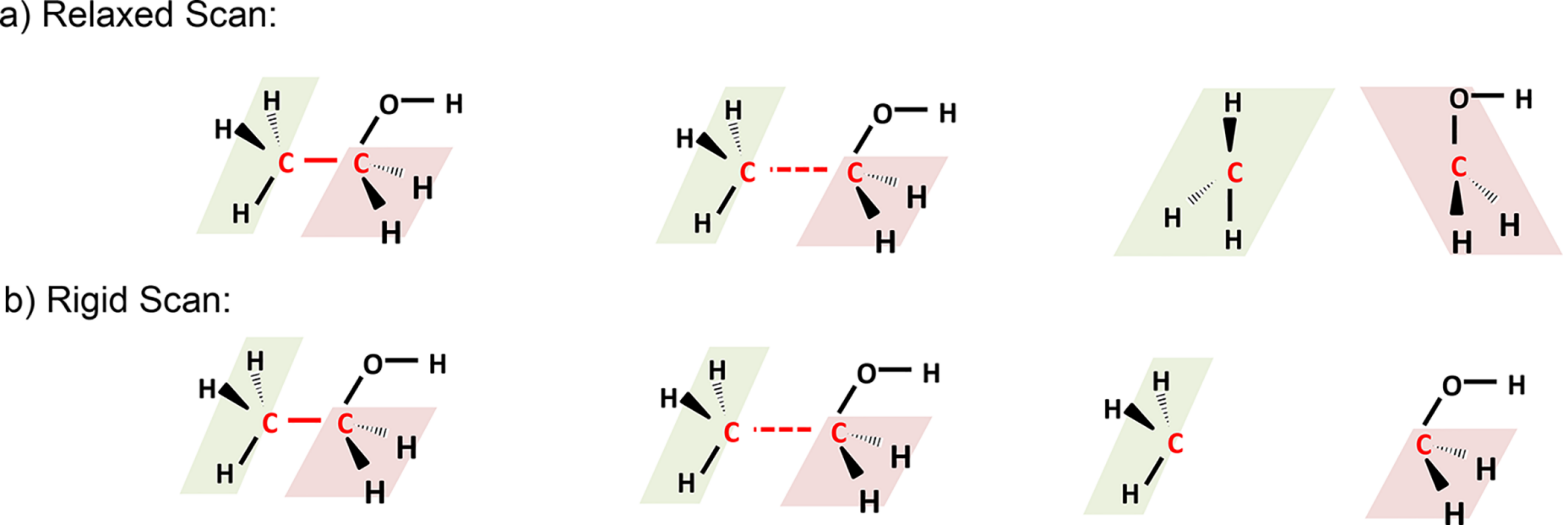
Illustration of (a) a relaxed carbon–carbon bond scan on ethanol and (b) a rigid carbon–carbon bond scan on ethanol.

The ScanPoint section also contains the “hasCalculation” Object property, which connects a “ScanPoint” instance to an instance of the “GaussianCalculation” class defined in OntoCompChem. This enables OntoPESScan to make use of the classes and relationships defined in OntoCompChem in addition to the data stored there and essentially means that each “ScanPoint” in a “PotentialEnergySurfaceScan” is associated with its own entry in OntoCompChem. Through OntoCompChem, properties such as the SCF energy shown in Figure 2 and the full three-dimensional geometry are available for each scan point, meaning these concepts and this data does not need to explicitly stored and duplicated in OntoPESScan. This link also helps distinguish between two commonly supported types of scans that can be performed using computational chemistry packages, namely, relaxed scans, where a full geometry optimization is carried out at each point along the scan, and rigid scans, where only the defined scan-coordinate is modified while other geometric coordinates are left unchanged where possible. These two types of scans are illustrated in Figure 4.

Figure 4 illustrates the difference between a relaxed and rigid scan for the carbon–carbon bond scan in ethanol. The fragments at a long distance have their geometries changed from when they are bonded together in ethanol, as a geometry optimization is performed for each point along the surface. In contrast, for a rigid scan, the bond length is elongated but the geometries of the fragments do not change. Notably, in terms of the scan coordinate, the atoms that define the scan coordinate are identical and the values of the scan coordinate can also be the same. However, they can be distinguished by differences in the electronic energy and of course the geometries along the surfaces will be substantially different, meaning such scans can be distinguished by leveraging the linked data in OntoCompChem. To facilitate this, a simple data property “hasInputAtomIDs” links a “ScanPoint” to a string that defines which atom indices in the input quantum chemistry calculation file were used to define the scan to make querying and finding information in the associated OntoCompChem entry simpler. Each “ScanPoint” having its own OntoCompChem calculation and InputAtomIDs also supports definitions of a potential energy surface scan from multiple different quantum chemistry jobs in addition to the cases where the scan is performed in a single job, providing flexibility.

### Population and Querying

Given the ontological structure, an ABox of OntoPESScan in the knowledge graph must also be accompanied by one ABox in OntoCompChem for every “ScanPoint” linked to the “PotentialEnergySurfaceScan” instance. To help with creating and uploading entries to the KG, a set of software agents have been developed to process output log files from a Gaussian program calculation and create the necessary OWL files for both the OntoPESScan and OntoCompChem instances, streamlining the population process. These are freely accessible at https://github.com/cambridge-cares/TheWorldAvatar. The OntoSpecies speciesIRI that the OntoPESScan entry is linked to provided as input as well so that the OntoPESScan instance in the KG is linked to both OntoCompChem and OntoSpecies at upload. This enables information in these ontologies to be utilized when querying OntoPESScan. For example, a federated query can be used to first search OntoSpecies for “Species” instances with an InChI that matches that of CO_2_ and then to search OntoPESScan for “PotentialEnergySurfaceScan” instances that have this species instance as the target of “onSpecies”. This essentially finds scans in the KG that were performed on CO_2_.
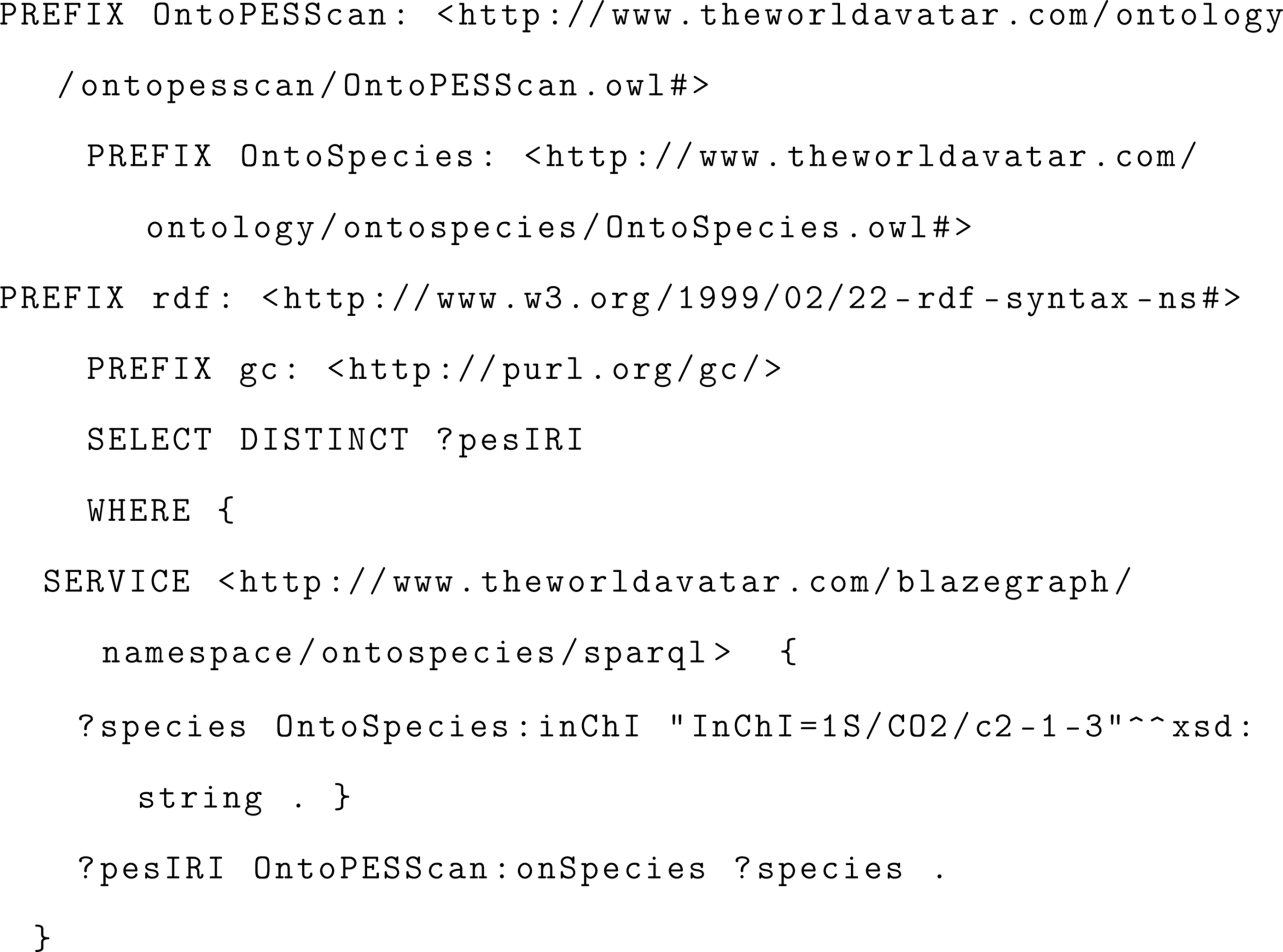


Similarly, a federated query can also be used to retrieve the electronic energies from OntoCompChem at each scan point for one of PotentialEnergySurfaceScan instances found in the previous query.
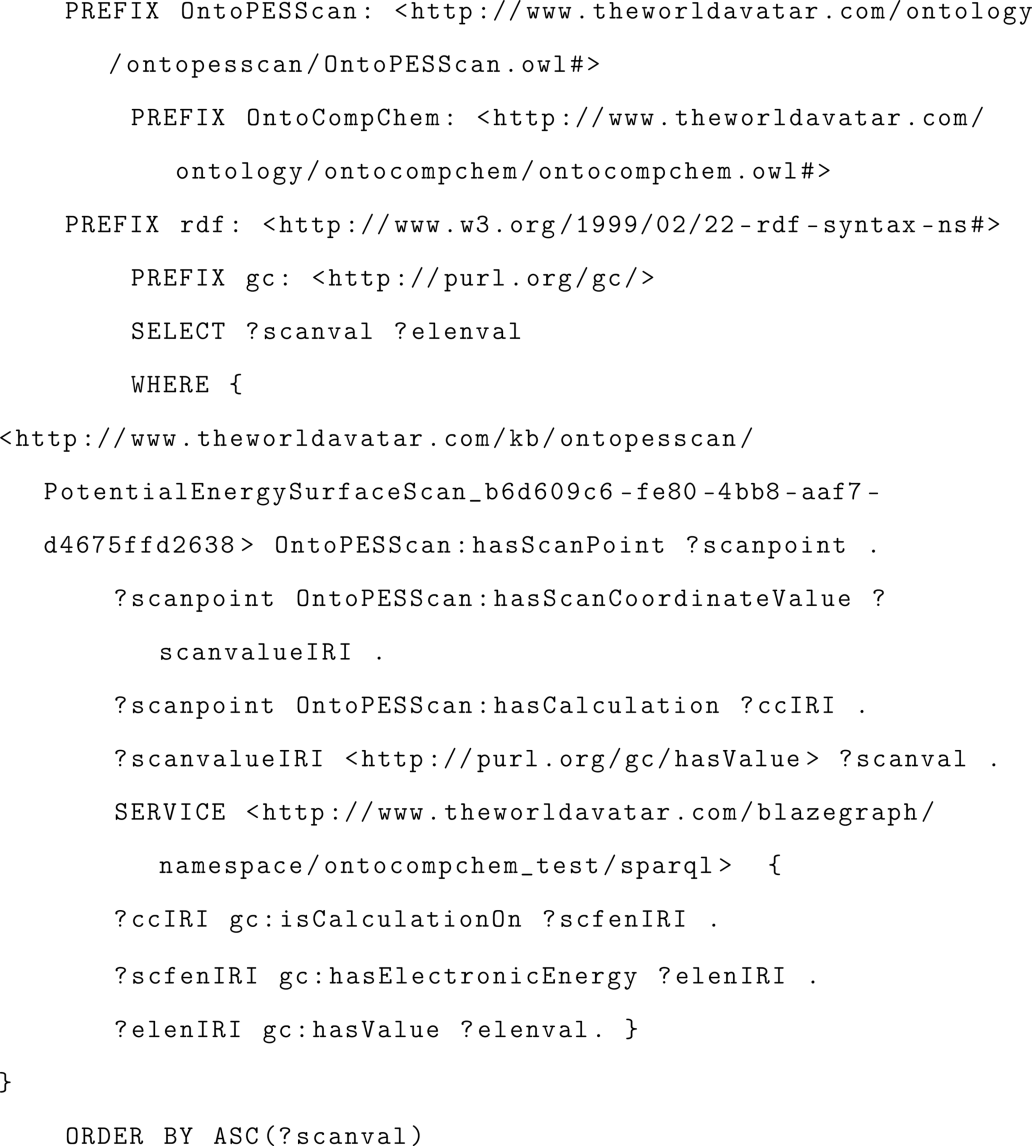


Overall, the OntoCompChem ontology is designed to concisely represent the key concepts and relations for scans on potential energy surfaces. It makes use of links to OntoSpecies to uniquely identify species in the scan and atoms in the scan coordinate and enables the user to query for any associated species properties, such as InChI, for example. OntoPESScan is also connected to OntoCompChem, so properties defined in OntoCompChem such as geometry or SCFEnergy are available for each scan point, which allows this information to be accessed without duplication among ontologies.

## Force-Field Fitting Agent

As mentioned previously, the determination and descriptions of potential energy surfaces find a wide variety of use in computational chemistry. This includes the accurate computation of rate coefficients of chemical reactions^2^ and the development of force fields for molecular dynamics (MD) simulations.^3^ Achieving this requires chemical data on potential energy surfaces, reactions, and the chemical species they describe.

The development of the OntoPESScan ontology is meant to facilitate the ease of automating the storage and retrieval of data related to potential energy surfaces that can then be utilized by various agents. In this section, we show how to benefit from the data stored in the KG to parametrize reactive force fields as a proof-of-concept. Future development will include using the data related to potential energy surfaces to calculate and improve rate coefficient estimates for chemical kinetic mechanism development. However, this will require highly accurate potential energy surfaces and the consideration of conformational states, which would require extensions to the ontology.

Designing reactive force fields for MD simulations is very challenging because it necessarily involves dealing with a multidimensional problem, where the interactions need to be modeled by highly complicated functional forms with many strongly coupled parameters that are optimized via a difficult search. Unfortunately, a general parametrization for the commonly used reactive force fields is not available yet; instead, parameters are tuned to specific chemical systems and environments. In this work, in order to avoid the complications of building reliable reactive force fields, we selected the empirical valence bond (EVB) force-field coupling method^50^ to join two classical force fields from literature, forming a truly reactive force field as a result. The main advantage of the EVB method when compared to reactive force fields is that the simulation of reactive processes is conducted via the coupling of nonreactive force fields that are already available in the literature to describe the chemically different states. Moreover, compared to the fitting of reactive force fields, which requires a large set of quantum mechanical structure and energy data, fitting the EVB force field only requires the potential energy surface for the reaction of interest to the study. In addition, despite the initial task of calibrating the coupling terms against reference data, research has demonstrated that these couplings are invariant to the surrounding electrostatics, making it possible to simulate the same reactive unit in different environments.^51^ These features of the EVB method have increased its recognition as a practical and reliable tool within the computational chemistry community.^50,52^ However, as soon as the KG is populated with more data, the fitting procedure developed in this work can be easily extended to calibrate other force fields and functional forms. Moreover, the ontology is developed in such a way that other methods for fitting surfaces such as neural networks could also make use of the information stored in our knowledge graph and act as agents.

In the EVB method, a classical force field is assigned to any different chemical state for the system. The EVB method defines a Hamiltonian whose matrix representation has each of the computed energies of the involved chemical states as diagonal components, whereas the off-diagonal terms are given by the coupling terms between the force fields in the reaction. Matrix diagonalization at each time step allows the computation of reactive energy landscapes that account for the change in chemistry when conformations between the participating chemically different states are sampled. More details on EVB theory are reported in the Supporting Information.

**Figure 5:**
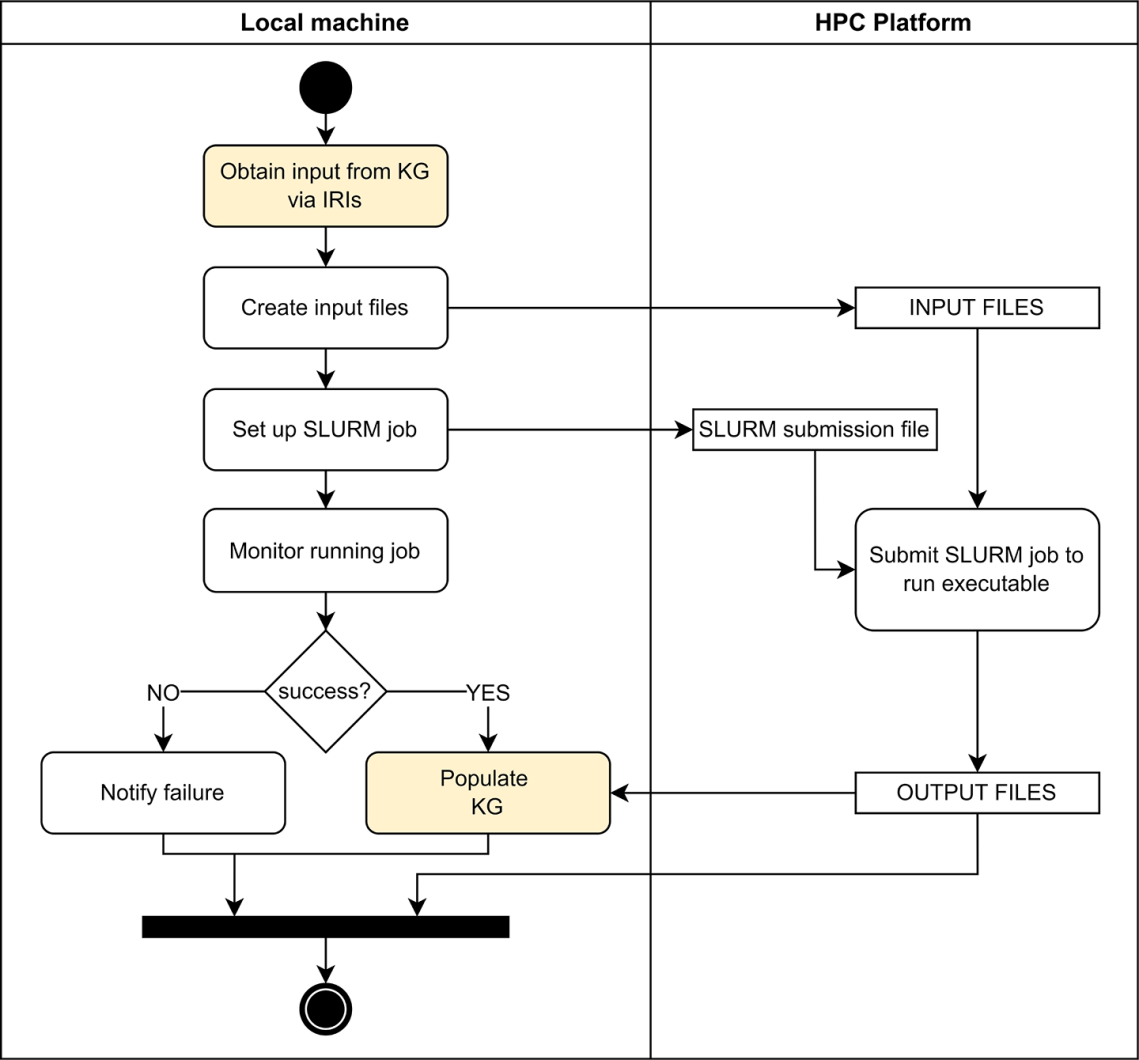
UML activity diagram of the force-field fitting agent. The agent enables the calibration job to be executed on a HPC platform. The yellow shaded actions indicates the data retrieving operation of agent over the knowledge graph and the knowledge-graph-populating operation.

The force field calibration is performed by an agent that follows the agent template designed by Mosbach et al.^31^ with few changes. A unified modeling language (UML) activity diagram of the force-field fitting agent is provided in Figure 5. The process starts by querying from the knowledge graph the information required by an executable that performs the force field calibration, giving as input OntoPESScan IRIs. SCF energies and geometries of each scan point are retrieved from the OntoCompChem instances linked to the OntoPESScan entry. The agent creates the input files in the format required by the executable and transfers them to the HPC platform. A SLURM job is set up and submitted to the HPC system. The job is then monitored using a status file associated with job. Finally, in the case of a successful run, the fitting parameters for the force field are then retrieved by the agent and added to the appropriate scan in the knowledge graph. In this work, the executable performs the EVB coupling term calibration. However, the force-field fitting agent is developed in such a way that its extension to other functional forms or other methods for fitting surfaces will require only the development of new executables to be submitted by the agent to the HPC platform with only a few minor changes to the main code of the force-field fitting agent.

The EVB executable is designed to accept the energies and geometries of each scan point as input to perform parameter estimation for the target force field. To achieve this, the EVB executable calculates the energies and adjusts the parameters within the target force field to replicate the potential energy surface. The EVB executable workflow covers different tasks that are performed with different software, which are detailed as follows:

1. The *XYZ* coordinates and the SCF energy of each scan point are given as input to the executable. A configuration file that contains information on the classic force field scheme to be used is also given as input.
2. States of the reactants and products are identified from the PES curve. Local minima energies in the PES and energy at the scan coordinate maximum distance (in case of bond scans with one local minima in the PES) are selected by the executable as reactant and product chemical states.
3. Classic force field are assigned to each state using DL_FIELD.^53^ The DL_FIELD tool converts a user’s atomic configuration in simple *XYZ* coordinates into identifiable atom types based on a particular user-selectable force field scheme that looks at the neighboring atoms for each atom in the system. All the force field information for each chemical state is stored in a topology file (DL_POLY format). In the case DL_FIELD is unable to assign the topology to one of the state, the agent notifies the job failure. In such circumstance, an user-defined topology for that state can be given as an input to the executable.
4. The two classic force fields energies for each geometry along the scan coordinate are calculated using DL_POLY.^54,55^
5. The EVB coupling term is calibrated using MoDS.^56^ MoDS is an integration of multiple tools developed for various generic model development tasks, such as parameter estimation,^26,57^ surrogate model creation,^58^ and experimental design.^59^ The calibration procedure is described in the Supporting Information.

## Results and Discussion

**Figure 6:**
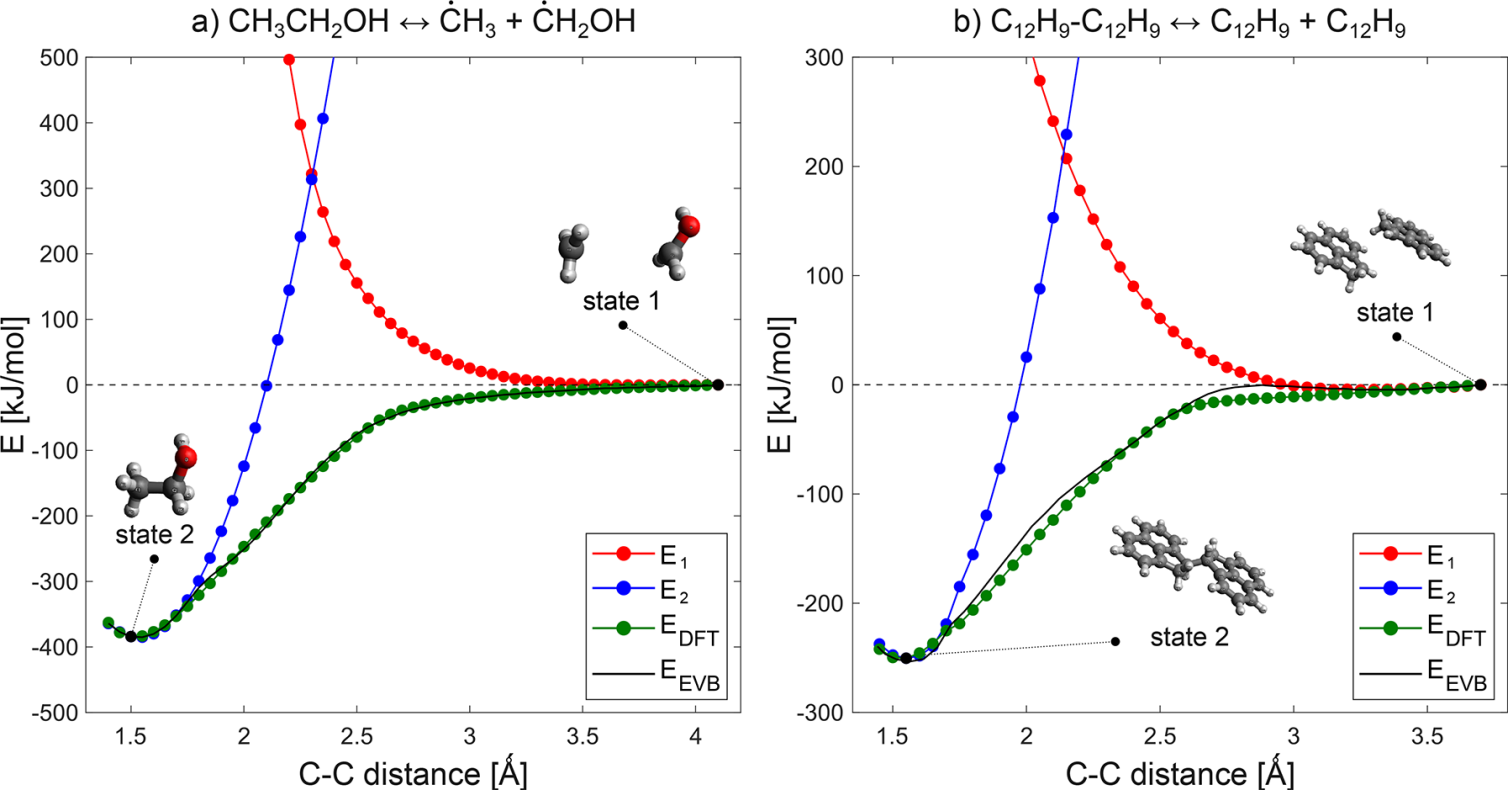
Energy profiles along (a) the C–C bond distance in ethanol and (b) the two π-radical sites in the 1,2-dihydroacenaphthylen-1-yl dimer formation. Green lines show the DFT energies used as references. The zero of the energy was chosen as the C–C maximum distance (state 1) for clarity. Red and blue curves show the energies calculated with the classic force field for state 1 and 2, respectively. Black curves show the obtained EVB potential energies.

We selected two use-cases as preliminary examples: the C–C bond scan in the ethanol molecule (Figure 6a) and 1,2-dihydroacenaphthylen-1-yl dimer formation (Figure 6b). The first case is selected to show that any bond breakage and formation can be accurately described using this methodology. The latter is a more interesting case that finds new applications in the combustion field. 1,2-Dihydroacenaphthylen-1-yl is a localized π-radical. Recently, quantum mechanics/molecular mechanics (QM/MM) simulations showed that π-radicals bond strongly enough for stability at flame temperature and react rapidly through physically stabilized internal rotors toward soot nanoparticles.^60^ However, current reactive force field parametrizations are unable to detect bond formation between two localized π-radical sites, so only ab initio or QM/MM approaches can be used to study such a system at the moment.

Figure 6 shows the energies profiles for the two selected cases. Relaxed potential energy surface scans were performedalong the C–C bond in the ethanol case and along the *pi*-two radical sites in the 1,2-dihydroacenaphthylen-1-yl dimer case using Gaussian 16.^41^ In both cases, the geometries were optimized at the B3LYP/cc-pVQZ level of theory and the energies were refined with single-point energy calculations at the M06-2X/cc-pVQZ level of theory. All DFT calculations were carried out using the spin-unrestricted formalism. It is important to note that both these surfaces involve association and dissociation between singlet states and doublet product states. As such, multireference methods would be more appropriate for the computation of these potential energy surfaces than the DFT methods employed here. However, DFT methods are much more tractable computationally, with the 1,2-dihydroacenaphthylen-1-yl dimer potential energy surface being too large to handle with multireference methods, so DFT was used for consistency between the two examples. As a check, the ethanol dissociation surface was computed with the multireference CASPT2 method using a two-electron, two-orbital active space and the cc-pVDZ basis set. This surface is compared to the B3LYP/cc-pVQZ surface in the Supporting Information. The overall agreement is good, suggesting that DFT methods can be applied for such potential energy surfaces. The EVB method fits the CASPT2 data well, suggesting that the procedure is applicable to a wide variety of computational chemistry calculations. The main focus is on the use of the data rather than the accuracy of the underlying PES, but it is noted that using the fittings from the agent does require accurate surfaces.

The obtained DFT energy profiles (black lines in Figure 6) were used as quantum chemical reference energies for the force filed calibration. Scan points corresponding to local energy minima and the maximum distance are selected as state 1 and state 2, respectively, by the executable. Force fields were generated with the DL_FIELD program using the OPLS2005^61,62^ force field library for the two different chemical states. In the ethanol case, DL_FIELD fails to create the topology for state 1 because it is unable to assign any atom type for carbon atoms that contain three coplanar bonds with noncarbon atoms. Therefore, in this case, a user-defined topology is given as input for the •CH_3_ and •CH_2_OH radicals. In the 1,2-dihydroacenaphthylen-1-yl case, where the OPLS2005 Lennard-Jones term is known to overestimate the dispersion energies, the isoPAHAP force field is used to describe the intermolecular interactions.^63^ At each scan point, energies are calculated with the two selected force fields using DL_POLY. Finally, the EVB coupling term is calibrated to fit the DFT energy profiles using MoDS. The EVB potential energies exhibit good agreement with the DFT reference data in both cases, with a maximum residual of 11 kJ/mol for the ethanol case and that of 14 kJ/mol for the 1,2-dihydroacenaphthylen-1-yl dimer case.

This methodology can be used to fit force fields able to describe any system in which the reactions of interest were previously identified and the obtained force field can be easily extended by adding new reactions and chemical states. The fitting parameters can then be used for a given MD application to describe the dynamics of chemical reactions as a valid alternative to more complex methods such as reactive force field and QM/MM methods. MD simulations are not reported here as they are outside of the scope of this work, but they will be part of future work.

## Conclusions and Outlook

In this work, a new ontology for the representation of exploration of potential energy surfaces, OntoPESScan has been developed. This ontology adds further support for the representation of computational chemistry concepts in The World Avatar. The OntoPESScan ontology makes use of linked data principles by containing relations that link to existing chemistry ontologies in OntoSpecies and OntoCompChem. This enables potential energy surfaces to be queried by species information in OntoSpecies and points along a potential energy surface to have their energies and geometries stored and described using concepts in OntoCompChem in a semantic way. Additionally, a force-field fitting agent has been developed to make use of the linked data in the OntoPESScan ontology and showcase the advantages of the knowledge graph. This agent shows how force fields for reactive systems can be parametrized on-the-fly by applying the empirical valence bond coupling method to potential energy surfaces utilizing the description of the surface in OntoPESScan in conjunction with the linked computational chemistry data in OntoCompChem. The agent was demonstrated for two potential energy surfaces. The first is a well-known surface corresponding to carbon–carbon bond scission in ethanol. The second corresponds to a newer case of reactions between localized π-radical polyaromatic hydrocarbons. In both cases, force fields were fitted for the reactions’ potential energy surfaces, which could then be used to perform molecular dynamics simulations.

Going forward, further development of OntoPESScan and other ontologies for chemistry is planned to extend the capabilities of The World Avatar. Development to support higher dimensionality representations of the potential energy surface will be key, as applications that require highly accurate potential energy surfaces and larger molecular systems are likely to require more than just 1D representations. A potential extension to this would be the development of an ontology and semantic representation of molecular dynamics simulations and results, as this is a key area of computational chemistry. Such an ontology for molecular dynamics would naturally link well with OntoPESScan, as the information on how force field fitting parameters for a given molecular dynamics simulations are derived would be found in OntoPESScan and OntoCompChem. Additionally, agents could use the information in OntoPESScan and OntoCompChem to fit new force fields on the fly for different molecular dynamics simulations, which could then be represented in the knowledge graph as well. As with molecular dynamics, potential energy surfaces are also key for computation of rate coefficients during mechanism development. Development and coupling of OntoKin or other ontologies for rate coefficients with OntoPESScan would also work toward agents being able to continuously calculate and improve rate coefficient estimates for chemical kinetic mechanism development. As rate coefficients require highly accurate potential energy surfaces, this would require development of OntoPESScan to represent more complex potential energy surfaces and coordinates as discussed above. This will all help work toward an open, continuous, and self-growing knowledge graph for chemistry.

## Data Availability

The World Avatar knowledge graph and the Force Field Fitting agent are publicly available on GitHub at https://github.com/cambridge-cares/TheWorldAvatar/ under the MIT license. The software agent uses third-party software. DL_FIELD and DL_POLY are available under an academic license (https://www.ccp5.ac.uk/software/), while MoDS and Gaussian 16 are commercial software available from https://cmclinnovations.com/ and https://gaussian.com/, respectively. Gaussian output files for ethanol and π-radical potential energy surfaces supporting this publication are also provided in the University of Cambridge data repository at DOI: 10.17863/CAM.82487.

## Supporting Information Available

Information on the empirical valence bond approach and the calibration of the empirical valence bond coupling term (PDF)
