# Supplementary material for: OntoPESScan: An Ontology for Potential Energy Surface Scans
Source: ACS Omega. 2023 Jan 3;8(2):2462–75. doi: 10.1021/acsomega.2c06948 (PMC9850739; doi:10.1021/acsomega.2c06948)
Supplement: Supplementary file 1 — ao2c06948_si_001.pdf [file ao2c06948_si_001.pdf]

Supporting Information: OntoPESScan: An Ontology for Potential Energy Surface Scans

Angiras Menon,[†] Laura Pascazio,[‡] Daniel Nurkowski,[¶] Feroz Farazi,[†] Sebastian Mosbach,[†,‡] Jethro Akroyd,[†,‡] and Markus Kraft[*,†,‡,§,‖]

†*Department of Chemical Engineering and Biotechnology, University of Cambridge, Philippa Fawcett Drive, Cambridge, CB3 0AS, UK*

‡*CARES, Cambridge Centre for Advanced Research and Education in Singapore, 1 Create Way, CREATE Tower, #05-05, Singapore, 138602*

¶*CMCL Innovations, Sheraton House, Castle Park, Cambridge CB3 0AX, UK*

§*School of Chemical and Biomedical Engineering, Nanyang Technological University, 62 Nanyang Drive, Singapore, 637459*

‖*The Alan Turing Institute,London, United Kingdom*

E-mail: mk306@cam.ac.uk

Appendix

Empirical Valence Bond Approach

The EVB approach describes chemical reactivity by taking into account the diabatic states corresponding to the classical valence-bond structures describing the reactant and product states of a reaction, and any intermediate states, if they exist. A simple schema for a two-state reaction is shown in Fig. S1, where the energy of each diabatic state is represented by a

non-reactive force field (FF), one non-reactive FF for the reactants (E_1) and one non-reactive FF for the product (E_2).

The EVB method defines a pseudo-Hamiltonian matrix ($H_{\mathrm{EVB}}(R)$) whose matrix representation (Eq. 1) has the potential energies of the reactant and product diabatic states at a given structure obtained from standard non-reactive force fields as diagonal components ($E_1(R)$ and $E_2(R)$), whereas the off-diagonal terms are given by the coupling C_{12} between the force fields in the reaction:

$$H_{\mathrm{EVB}} = \begin{pmatrix} E_1(R) + \epsilon_1 & C_{12}(R) \\ C_{12}(R) & E_2(R) + \epsilon_2 \end{pmatrix} \tag{1}$$

where all terms depend on the set of atomic coordinates R. The ϵ_1 and ϵ_2 values in Eq. 1 are constant diagonal energy shifts, usually chosen so as to reproduce the known exo or endo-thermicity of the reaction in question.

Following diagonalization of $H_{\mathrm{EVB}}(R)$, we obtain two possible eigenvalues, $\lambda^{\pm}$,

$$\lambda^{\pm} = \frac{1}{2}\left[E_1(R) + E_2(R) \pm \sqrt{(E_1(R) + E_2(R))^2 - 4\left[E_1(R)E_2(R) - C_{12}^2(R)\right]}\right]. \tag{2}$$

The EVB energy (E_{EVB}) is defined as the lowest eigenvalue:

$$E_{\mathrm{EVB}} \equiv min(\lambda^+, \lambda^-) \tag{3}$$

The off-diagonal coupling elements C_{12} is typically a Gaussian function of the set of atomic coordinates R. For the implementation of the EVB method in DL_POLY, C_{12} has the following functional form:

$$C_{12} = A_1 \exp - \left(\frac{E_1(R) - E_2(R) - A_2}{A_3}\right)^2 + A_4 \tag{4}$$

To obtain an EVB potential, the diagonal force fields are first compared to ab initio results (or experiment) ((E_{ref}) in Fig. S1) for the corresponding stable species, and if necessary, small adjustments are made in order to reproduce the structures. Then, the behavior of the diagonal force fields (shifted appropriately to take reaction energy into account) along the reaction coordinate is assessed, and compared to ab initio data.

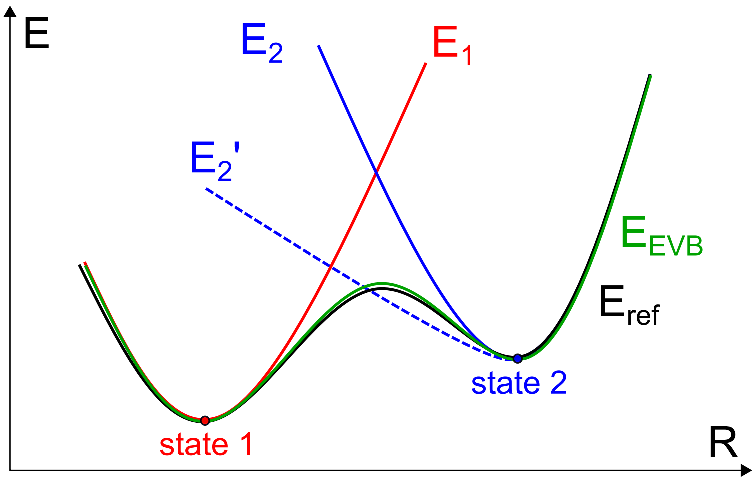

Figure S1: Schematic representation of an EVB reactive potential energy surface, E_{EVB} (solid green line), obtained from two diabatic states E_1 (solid red line) and E_2 (solid blue line) corresponding to reactant and product states. Reference energy landscape is shown in black. An example of an inappropriate product diabatic state function, E_2' (dashed blue line), that lies lower than the target potential energy for some values of R, is also shown.

As shown in Fig. S1, if we used the force field built for state 1 (E_1), state 2 will never be sampled, since the values of E_1 in the region of state 2 will be exceedingly large. Analogously, state 1 will never be sampled if we used E_2 as the force field to describe the interactions. The effect of diagonalization of the EVB pseudo-Hamiltonian is and can only be to lower the energy relative to the lowest of the diagonal state energies. Hence if one or more of the diagonal states is significantly lower in relative energy than the ab initio data (E_{ref}) in one section of the reaction coordinate, as for the case of E_2' shown in the schematic Fig. S1, then it will be impossible to get a good fit of the potential energy surface.

Calibration of EVB coupling term

Once it has been established that the diagonal force fields E_1 and E_2 produce an acceptable description, the off-diagonal terms (C_{12}) can be fitted to the target potential energy surface E_{ref}. The set of parameters in Eq. 4

$$\theta = (A_1, A_2, A_3, A_4)$$

is estimated to minimize the least-squares objective function given by:

$$\Phi(\theta) = \sum_{n=1}^{N} [E_{\text{EVB}}(R^{(n)}, \theta) - E_{\text{ref}}(R^{(n)})]^2 \tag{5}$$

where E_{EVB} is given by Eq. 3, E_{ref} is the the reference energy landscape (usually ab initio data), N is the number of scan points and R is the set of atomic coordinates. The calibration process initially employs low-discrepancy quasi-random global sampling through a Sobol sequence generator[S1]. This provide intial points for a SolvOpt optimization algorithm[S2], selected for the non-linearity of the least-squares objective function.

Ethanol dissociation surface

The ethanol dissociation surface was computed with the multi-reference CASPT2 method using a 2-electron, 2-orbital active space and the cc-pVDZ basis set. This surface is compared to the B3LYP/cc-pVQZ surface in Fig. S2a . The overall agreement is good, suggesting that DFT methods can be applicable for such potential energy surfaces. The EVB method fits the CASPT2 data well (Fig. S2b, suggesting that the procedure is applicable to a wide variety of computational chemistry calculations.

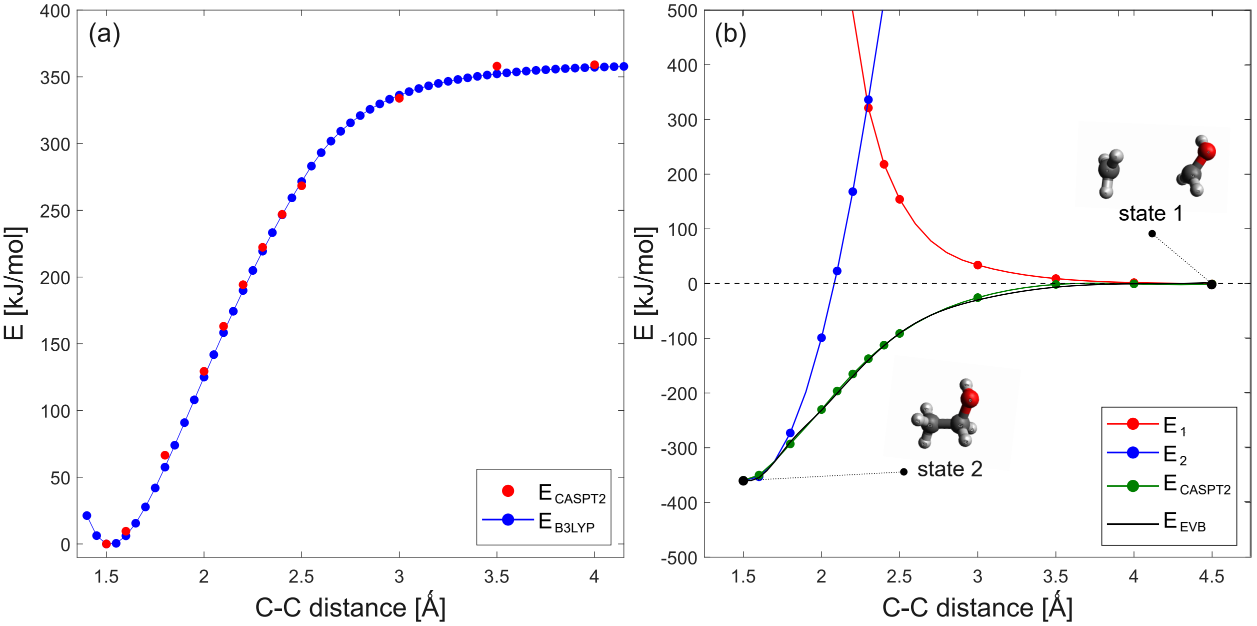

Figure S2: (a) Comparison between the dissociation surfaces along the C-C bond distance in ethanol using the multi-reference CASPT2 method with cc-pVDZ basis set and the B3LYP/cc-pVQZ DFT method. (b) Energy profiles along the C-C bond distance in ethanol. The green line shows the CASPT2 energies used as reference. The zero of energy was chosen for clarity as the CC maximum distance (state 1). Red and blue curve show the energies calculated with the classic force field for state 1 and 2 respectively. The black curve show the obtained EVB potential energies.

References

(S1) Sobol, I. On the systematic search in a hypercube. *SIAM Journal on Numerical Analysis* **1979**, *16*, 790–793.

(S2) Shor, N. Z. *Gradient-type Methods with Space Dilation, Minimization Methods for Non-Differentiable Functions*; Springer-Verlag, New York, 1985; pp 3:48–92.
